# The effects of forest therapy on public mental health and circular economy: A policy support model in Japan

**DOI:** 10.3389/fpubh.2022.1042589

**Published:** 2022-10-28

**Authors:** Shujian Zhang, Junfei Teng, Yan Zeng, Honglin Song, Zhijun Gu

**Affiliations:** School of Government, Shenzhen University, Shenzhen, China

**Keywords:** public mental health, Circular economy, Forest therapy, MBPS, policy support, Japan

## Abstract

**Background:**

Forest therapy has gained popularity in Japan and even other nations/regions due to its health benefits. In addition, forest therapy has contributed to the development of circular economy and industrial upgrading. Japanese successful practice can serve as a model for other countries in the Asia-Pacific region. To this end, the aim of this study was to determine whether forest therapy can improve the whole well-being of the participants and has a positive effect on the development of circular economy in the region.

**Methods:**

Both empirical and inductive research methods were used; empirical approach was conducted to perform comparative analysis of regional data that was retrieved from the research project of Japanese Forestry Agency in 2015. Specifically, the efficacy of forest therapy on physical (blood glucose, blood pressure, body weight) and mental (sleep quality e.g.,) health outcomes among 815 participants was investigated. Regional data are from the statistics of Iiyama City from 1990 to 2005. After the concept of forest therapy became popular in the late 1990s, this element had a great positive impact on the economic benefits of Ishiyama City and other major forest scenic areas. We summarize and analyze a series of policies made by relevant departments of the Japanese government in the years from 2019 to 2021 to promote forest therapy and related circular industry development.

**Results:**

Significant (pre-to-post participation) changes in physical measure was observed. Firstly, mean weight of those overweight participants decreased across three different time points (pre-test/enrollment = 79.7 kg, 3-month participation = 77.2, and 6-month participation = 76.8 kg), while overall mean weight of the participants decreased to 61, 60.5, and 60.4 kg, respectively. Secondly, Participant with normal weight showed a decrease on mean HbA1C (from 6.09 to 6.06) at Week 24, while overweight participants demonstrated a slight change 6.03–6.01 after 6 months the average HOMA-IR for overweight participants decreased from 3.5 to 2.5 at Week 24, while participants with normal weight demonstrated a decrease from 2.2 to 1.7 at Week 24. Forest Therapy has emerged in Japan since Mid-1990s and has attracted a large number of tourists all over the world due to its unique health benefits.

**Conclusion:**

Forest therapy in Japan has positive effects on whole well-being of Japanese residents and it has helped public mental health promotion and economic growth. Under the guidance and support of government policies, it can promote the development of circular economy and industrial transformation and set a model of Japanese forest therapy development for other countries in the Asia-Pacific region.

## Introduction

### The gap between global environmental degradation and rising health expectations

With rapid economic growth over half century, the ecological environment has been severely damaged due to excessive use of natural resources and excessive emissions of carbon dioxide. Such environmental change has not only resulted in physical and mental health issues but also affected global sustainable development. Specifically, living environment becomes worse as the economic development continues, which is not the ideal way of life (1). Deterioration of ecological environment is one of the greatest challenges for humanity as the temperature of the earth's surface continues to rise (2). If developed economies do not lead the world on a sustainable development path, human health will continue to suffer from environmental pollution (3). As the earth's environment has become irreversibly worse than before the industrial era, the generations between the 1960s and 2020s will be more exposed to the effects of extreme climate events, and their living environment will be significantly worse (4). This means that modern people are more prone to the decline of immunity resulting frum environmental problems, making human health problems more serious (5, 6). The pace of modern society is fast, and people generally feel that life is too stressful, which can easily lead to the deterioration of people's health (7). The approach to this problem must be pluralistic as health goals and biobased-economy development paths can only be considered in conjunction with better public policy (8).

From late 2019, human health issues (like depression, anxiety, sleep problem, increased sedentary behavior, and physical inactivity) are much more complicated than ever due to the COVID-19 pandemic (9–14). People's health has been affected by various social pressures, which have been exacerbated by the pandemic that has been ravaging the world for 3 years (15–20). The pandemic does not only dealt a fatal blow to human beings physically (20–23), but also caused great economic and mental pressure due to the prolonged lockdown (24–29). Only in the first year of the COVID-19 pandemic, global prevalence of anxiety and depression increased by a massive 25%, according to a scientific brief released by the World Health Organization (WHO) (30). The unprecedented stress has been caused by the social isolation resulting from the pandemic (31–33). All age groups with health and clinical conditions reported different levels of mental health issues because of the pandemic (34–41). Even if some people are allowed to travel, they must endure long quarantines at their destination hotels. These restraints have had a serious negative impact on people's health (42).

### Japanese forest therapy as a classic mindfulness-based practices

Regular engagement in physical activity is an important way to maintain well-being (43–47). Within an umbrella of physical activity, mindfulness-based Practices (MBPs) as traditional Eastern approaches have a greater emphasis on coordinative slow movement and deep breathing technique in line with meditative state (48–51). Such unique (easy-to-learn and safe) features have attracted significant number of practitioners (including those with chronic illness) (52–57) because its positive effects on relieving stress-related negative emotion and rehabilitative outcomes. MBPs play an increasingly important role in health and circular economy promotion, especially in a policy environment where people are under pressure to reduce carbon emissions.

Forest therapy in Japan is at the forefront of MBPs as a natural health treatment (58). Japan's vision of the effects of mindfulness is not limited to a single aspect. Forest therapy is an important way to bring together mindful exercise, mental health, and forest circular economy, as the idea of circular development has a very good historical and policy foundation in Japan, and it integrates comprehensive cognition of medicine, health science, economics, and public policy (59). The aim of this study was to analyze the reason how Japan makes forest therapy and the circular economy promote each other from the perspective of public policy and study the way how Japan combines the forest resources and the MBPs, encourages people to live in harmony with nature, better improving national health level (60). As the idea of ESG (Environmental, Social and Governance factors to evaluate companies and countries on how far advanced they are with sustainability) gradually prevails in the global development, the Japanese government firmly increases the investment and protection of forest resources through various policies, which has laid a good policy foundation for encouraging people to go into the forest and carry out MBPs treatment. Especially in the post-COVID-19 era, Japanese governments at all levels have reached a strong consensus on the coordinated development trend of forest therapy industry and circular economy in the face of great challenges posed by long COVID syndrome.

### Japan focuses on the multiple functions of forest resources

Japan's forest coverage is 68.5 percent, and its forest service industry policy is also quite comprehensive and detailed. Japan attaches great importance to the dual benefits of forest industry for people's health, well-being and the circular economy of the society and has set up forest therapy bases throughout the country since 1997 (61, 62). These specific projects are undertaken by local governments, medical groups, tourism bases and other groups. Many local governments would take forest resources into account when they launch regional development policies (63). Forest resources will play an even greater role in economic and health recovery in the post-pandemic period, especially after the novel Coronavirus impact and the mental health problems caused by the prolonged lockdown.

Forest therapy encourages enterprises and local governments to maintain and use forest resources under good planning. In the use of forest resources at the same time, a large number of people will be directed to the forest, and then the forest function and economic development combined, which is more in line with the concept of circular development in Japan. It is obvious that the market mechanism will not spontaneously adjust to this goal. The government needs to use policy tools to guide local governments and enterprises to see the advantages of combining the dual functions of forest resources for health care and circular economy. Such a development philosophy would reduce the destruction of forest resources by local governments and curb their urge to develop polluting industries (64). In recent years, forest therapy and its estimated preventive effect are attracting more and more attention (65). Facing the great pressure of carbon neutrality, the world should seriously consider the positive effects of circular economy on human beings (66). Japanese forest therapy is going global as many other countries start to accept the MBPs effectiveness of the forest therapy (67). In the era of global bio economy, major economies have developed many financial products related to sustainable development (68, 69). Japan's forest health industry and circular economy model are worthy of reference by the international financial community to better play the role of ESG in sustainable development.

## Materials and analysis

### Forest therapy as an MBPs way for national health promotion

**Figure 1:**
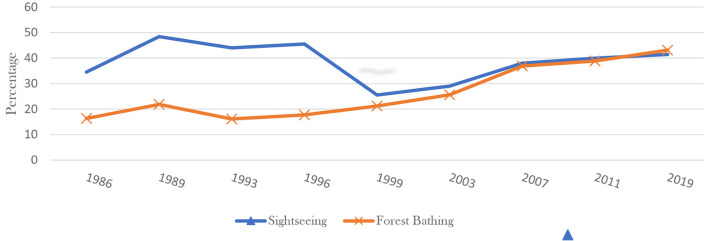
Forest bathing accounts for the percentage of people who go to the forest.

Japan has the longest life expectancy in the world and is also a country rich in forest resources. In mindfulness therapy, one of the main contents is to regulate mental state and reduce mental stress through meditation. From the perspective of the relationship between Japanese traditional culture and mindfulness meditation, mindfulness refers to spiritual baptism, which means that one can get spiritual sublimation. When conducting forest bathing, many activities can also be defined as MBPs and become part of forest therapy. From the perspective of participants, MBPs plays a regulating role in all aspects of the practice of forest therapy participants, by releasing the mental pressure and anxiety of participants, transfer their attention, reduce the psychological burden caused by the body and mind, so as to make their actions more natural and healthier (70). The Japanese place great emphasis on improving their health by exercising in the forest (71, 72). A continuing survey of the Cabinet Office, Government of Japan from 1986 released around every 3 or 4 years has revealed Japanese people's enthusiasm for forest bathing. The data indicates that Forest Bathing therapy as the purpose of visitors in forests has always ranked second (Figure 1) and even turned to get the first position in 2019. The upward trend that people go to forests for enjoying forest bathing has continued since the early 1990s.

**Figure 2:**
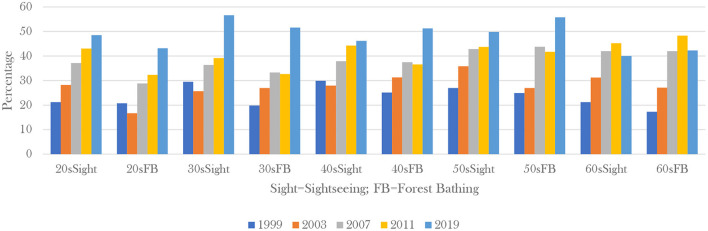
Comparison of the share of visitors in forests with sightseeing and forest bathing. ^*^In Japan, forest bathing is not only for the purpose of medical care, but also closely combined with tourism. Japan has one of the highest population densities in the world. For many Japanese people, the forest is one of the main places to release the pressure of life and adjust their emotions, so the activity of forest bathing is often with the purpose of leisure play (73).

The relationship between forest therapy and people's pursuit of health is even more evident when we compare specific data on visitors of different age groups. We can tell people under 40s year-level are more interested in sightseeing in forests while the purpose of forest bathing therapy of people over 40 almost surpassed the youth generations (Figure 2) in all surveys (73). Young people have no health pressure, so sightseeing is the primary purpose of visiting the forest. The fact that people over 40 years of age chose forest bathing as their first choice for forest activities shows the impact of forest therapy on health. Different from the Western view of life and death, East Asian cultural traditions place special emphasis on health, longevity and harmony between nature and human-being. Especially in Japan, a country that attaches great importance to the harmonious coexistence between nature and human-being, many elderly people see forest therapy as a way to bring them closer to nature and maintain physical and mental health. Evidence has shown that forest therapy can indeed keep the body functioning in balance, so forest therapy has been recognized and accepted by more and more Japanese people, especially the elderly (61).

### Data analysis of specific case-group from Japanese national forest therapy bases

Ministry of Health, Labor, and Welfare (MHLW) and Ministry of Agriculture, Forestry and Fisheries (MAFF) of Japan have jointly launched a forest therapy program, names as Smart Life Stay (SLS) from 2016, which aims to help improve health of the people who have higher blood glucose through forest bathing therapy. According to the data from MHLW, there are more than 11 million people whose HbA1c value is higher than 6%, 6.8 million's HbA1c value is higher than 6.5% and 2.7 million confirmed diabetics cases in Japan. The Japanese government set up 23 pilot forest therapy bases mainly in Honshu Island and Kyushu Island. The program typically consists of several consecutive days of forest therapy trips, during which visitors are instructed in specific diet management, exercise management and forest bathing activities. Physical data were compared before and after the program, and two more tests were conducted at 3 and 6 months after the program ended to confirm the effects of forest therapy on the health of the visitors. In addition, participants were also compared with many random non-participants.

**Figure 3:**
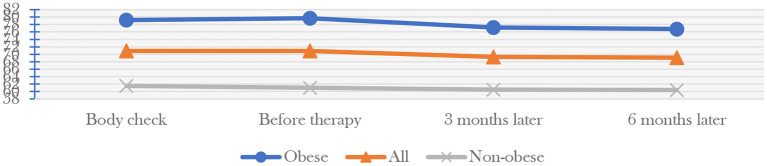
Weight Effect on participants after the forest therapy activity.

MHLW released the data of total 815 participants of the program in 2015 (Table 1), including 570 males between 50 and 55 years old and 245 females between 55 and 59. It was clear that most of the participants had health problems, particularly regarding hyperlipidemia and hyperglycemia, most of them had problems. 76% percent of the male participants were obese. The participants' weight fell (Figure 3) from the two medical examinations 3 and 6 months after the treatment. Obese people lost the most weight, from 79.7 to 77.2 kg after 3 months and 76.8 kg after 6 months on average. The corresponding figures for all participants on average are 70.9, 69.3, and 69.1 kg, for non-obese participants are, respectively 61, 60.5, and 60.4 kg. More than 98% of the participants were satisfied with the program's activities finally.

**Table 1:** Health condition of all participants in SLS program in 2016.

**Basic physical condition of 815 participants in 2016 SLS**					
**Health index**	**Category**	**Male**		**Female**	
Obesity	BMI (kg/m^2^)	58.6	76%	35.3	41.60%
	Waistline (cm)	71.8		30.2	
Blood pressure	Systolic (mmHg)	38.6	46.70%	30.2	33.90%
	Diastolic (mmHg)	35.1		13.5	
Blood lipid	HDL-C (mg/dl)	9.8	76.30%	2.4	71.80%
	LDL-C (mg/dl)	61.4		67.3	
Blood glucose	FPG (mg/dl)	48.8	85.10%	29	84.90%
	HbAlc (%) (NGSP)	78.2		80.8	

Data Source: Ministry of health, labor and welfare of Japan.

**Figure 4:**
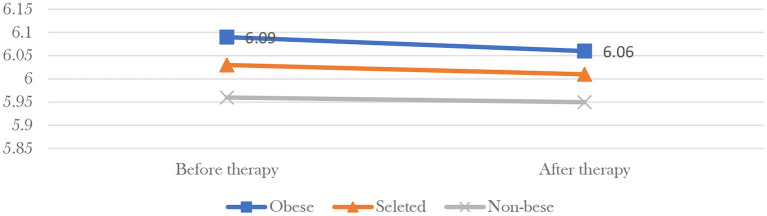
HbA1c value change of selected participants at four pilot bases. * HbA1c is a term commonly used in relation to diabetes. The term HbA1c refers to glycated hemoglobin. HbA1c is a product of glucose bound to hemoglobin in red blood cells. To a certain extent, the higher the blood glucose, the longer the duration, the higher the value, which can reflect the average blood glucose level in the past 2–3 months and has been used as the gold standard to evaluate the long-term blood glucose control in clinical practice. The normal reference value of the standard HbA1c detection method is 4–6%. Generally, diabetes requires HbA1c value below 7%, and >7% means an increased risk of diabetic complications. It develops when hemoglobin, a protein within red blood cells that carries oxygen throughout the body, joins with glucose in the blood, becoming “glycated”. In Japan, 6% proposed by the Japanese Diabetes Society in 2013 is generally used as the baseline point of treatment, and the goal is to keep the blood glucose value below 6% when people with diabetes receive relevant treatment. When the blood glucose level is between 6 and 7%, it means that attention should be paid to whether there are complications during treatment. When the blood glucose level is between 7 and 8%, it means that the condition of diabetes has become more serious.

**Figure 5:**
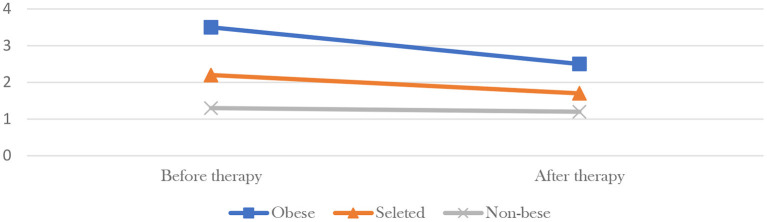
HOMA-IR change of selected participants in Aichi Prefecture pilot base.

Considering that 85 percent of all participants had hyperglycemia, the government used data from 195 randomly selected participants at four pilot bases (Figure 4) to show that the HbA1c (form of hemoglobin that is chemically linked to glucose) value of obese participants dropped from 6.09 before participation to 6.06, 6 months later, the non-obese from 5.96 to 5.95, and the overall mean from 6.03 to 6.01. The government also selected 118 participants from Aichi Prefecture to compare the change of HOMA-IR (important indicator of insulin resistance) value before and after the therapy (Figure 5), found the value of obese participants dropped from 3.5 before participation to 2.5, 6 months later, the non-obese from 1.3 to 1.2, and the overall mean from 2.2 to 1.7. Finally, data from all participants were compared with a randomly selected control group of non-participants and the effect was found to be significant.

#### Case of Iiyama city, Nagano prefecture

Policy support for forest therapy bases requires the efforts of local governments. For the forest base to operate for a long time and benefit residents and tourists, it must obtain the policy support from all aspects of the government. Two cities in Nagano prefecture, Iiyama and Saku, have been designated as forest therapy areas (74). Located in the northern part of Nagano Prefecture and backed by the Madarao Plateau, Iiyama city is rich in natural resources such as forests, lakes, and hot springs, and has been a good health resort since ancient times. Especially after the emergence of the concept of forest bathing, as one of the major cities in Japan close to forests and mountains, many tourists come to the city for forest therapy every year. The number of tourists is increasing year by year, and the forest service industry is booming, which opens a new path for tourism and circular economy in Iiyama city. Compared to the other cities, the forest area of Iiyama city is vast, providing visitors with good forest trails for healthy exercise and relaxation.

**Figure 6:**
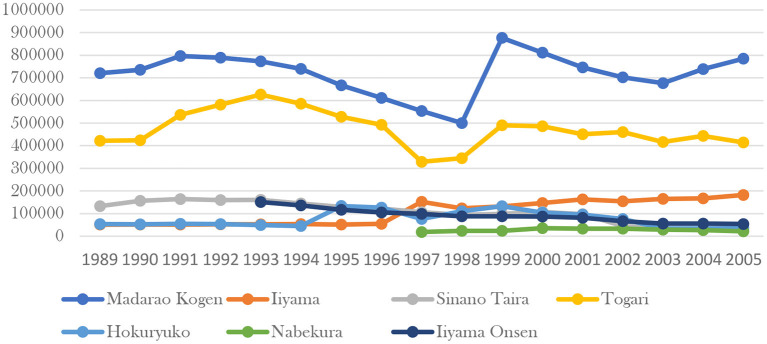
Comparison of tourist data of scenic spots in Iiyama city.

**Figure 7:**
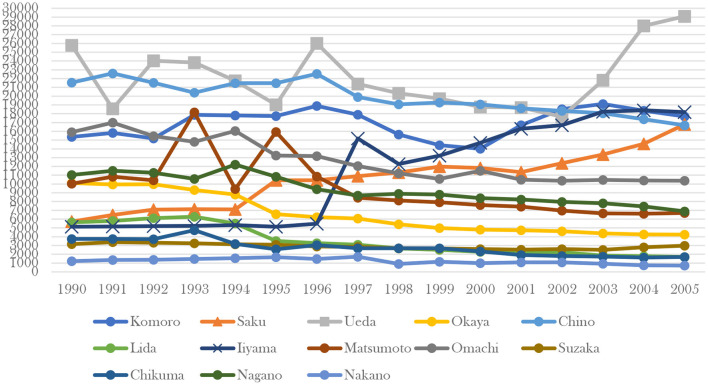
Comparison of tourist data of cities in Iiyama city.

Iiyama is an excellent sample city to test the effectiveness of forest therapy policy support. Some areas of the city are forest bathing pilot bases, while others are not. In Nagano Prefecture, Iiyama and Saku are the only two designated cities as forest therapy area. Iiyama city has seven main areas for tourists, including Madarao Kogen, Hokuryuko, Nabekura, Iiyama, Sinano Taira, Iiyama Onsen, Togari. From 1997, Iiyama and Madarao have been designated as forest bathing base under Iiyama city government's policy guideline. The tourist data (Figure 6) from 1989 to 2005 in these areas show Madarao Kogen and Iiyama have apparently seen an increase in tourist numbers since the new policy was introduced in 1997, and the trend has continued into the 21st century. In other areas the trend is in the opposite direction. If we observe the data (Figure 7) of same level as Iiyama city in the whole Nagano Prefecture, only Iiyama city, Saku city and Ueda city had obvious upward trend in tourists after 1997. Iiyama and Saku are the only two designated forest therapy cities in Nagano and the reason why Ueda also got a surge in tourist after 2002 was that the city of Ueda had built up more brand-new resorts since 2000.

#### Policy agenda

Faced with the increasing pressure of work and life and the serious mental problems, the Japanese society increasingly calls for attaching importance to forest and health, and the original concept of forest bath has received further attention and promotion (75). To promote industrial transformation and resource integration, realize the coexistence of natural resources and social development, and form a good economic cycle model, the Japanese government began to integrate the original concept of forest bath into the forest therapy industry, to promote the development of forest service industry. Since 2019, the Japanese government established the forest service review committee, led by the Forestry Agency, combined the main government departments to make joint industry associated support policy and the construction of the administrative system, hope to be able to further rational development of forest resources, in the health care and natural protection, and achieve a balance between social and economic development (76).

**Figure 8:**
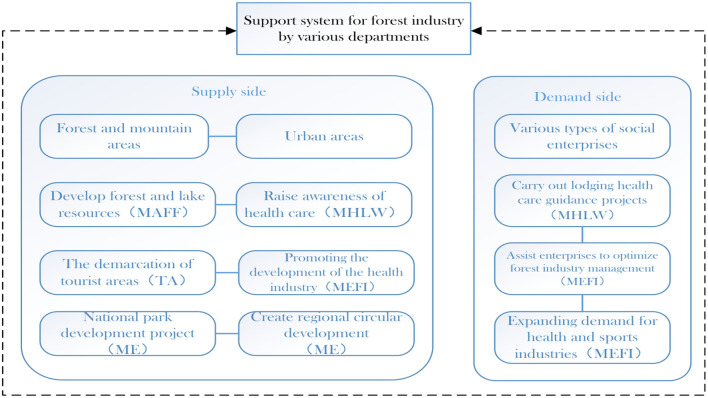
Japanese government's policy process on forest service resource integration.

In the industrial support system, the Japanese government starts from the supply side and the demand side (Figure 8) to integrate the forest service resources. On the supply side, the Ministry of Agriculture, Forestry and Fisheries (MAFF), which is responsible for the management of natural resources including forests and lakes, plays a leading role in promoting prominent areas with rich forest and lake resources and building designated bases for forest therapy. The Tourism Agency (TA) and the Small and Medium Enterprise Agency (SMEA) play an important role in the forest service industry, which is closely related to tourism and the revitalization of local economies. Health care is also an important part of the forest service industry. The Ministry of Health, Labor, and Welfare (MHLW) is responsible for the construction and cooperative development of related health care facilities, and the Ministry of Economy, Trade, and Industry (MEFI) further promotes the development of health care industry on this basis. The Ministry of Environment (ME) is also an indispensable part of the support system, as green and circular is one of the important characteristics of forest service. From the perspective of sustainable social and economic development, the Ministry of Health, Labor and the Ministry of Economy, Trade and Industry have integrated forest service with health care industry, responding to the concerns of The Japanese society on the theme of nature and health and striving to form a green circular economy in which forest service plays an important role.

## Results and discussion

### The health and economic effects of forest mindfulness therapy

From the perspective of the relationship between Japanese traditional culture and mindfulness meditation, mindfulness refers to spiritual baptism, which means that one can get spiritual sublimation. When conducting forest bathing, many activities can also be defined as MBPs and become part of forest therapy. From the perspective of participants, MBPs plays a regulating role in all aspects of the practice of forest therapy participants, by releasing the mental pressure and anxiety of participants, transfer their attention, reduce the psychological burden caused by the body and mind, so as to make their actions more natural and healthier (70).

Mindfulness therapy has many effects on human health. The first is diet management. According to the health survey data of the Ministry of Health, Labor and Welfare, many Japanese people have formed abnormal eating habits due to the mental stress of years of work, which affects the normal operation of the digestive system. Forest therapy can put participants in a less stressful surroundings, lower blood pressure, and encourage participants to self-correct their eating habits to prevent digestive diseases (77). Secondly, forest therapy is a process of “combination of static and dynamic”. In addition to meditation, jogging, climbing, and other exercises are also carried out in the forest environment. Compared with the urban environment and drug dependence, the exercise efficiency can be improved better, that is, the frequency of oxygen inhalation and cardiopulmonary exercise are more regular under the same exercise intensity (78). With a sound policy base and widespread support at the national and local levels, forest mindfulness therapy in Japan has achieved multiple effects in promoting health and a circular economy. From the observation group data released by MHLW in 2015 from national forest therapy bases, most participants, especially obese group, got health returns. Their weight, blood pressure, blood lipid, and blood glucose levels have gone down significantly 3 months after they finish the forest bathing project and even after 6 months all the values still have a minor falling. Data from a sample of people whose blood glucose levels were measured showed that forest bathing had a significant effect on the health of obese people.

From the local tourism economic data, the positive effect of forest therapy base policy is also obvious. In Nagano Prefecture, Iiyama and Saku are the only cities with forest therapy policies, and the number of tourists in these two cities has continued to increase since the introduction of the forest therapy base policy. In Iiyama city, there are totally ten tourist resorts. Iiyama and Madarao are the only designated forest therapy bases, and the number of tourists has skyrocketed in these two places since the introduction of the forest therapy base policy in 1997, while the number of tourists in other places has been declining year after year.

The close cooperation of relevant policy departments of the Japanese government has provided a good environment for the development of the mindfulness movement. The government departments of economy, environment and health have cooperated and issued some policies, which play a key role in the cultivation of local forest therapy bases. This virtuous policy cycle among the government, the base and the public is of great benefit to the development of the Mindfulness-based Practices movement in Japan. Meanwhile, the MBPs also promotes the health of Japanese citizens and the circular economy of the country.

### Policy support related to local development and circular economy

Japanese society attaches importance to the concept of forest and health, and the Japanese government also believes that industrial innovation should be carried out to build green circular development (79). Through consistent policy support and guidance from the central and local governments, forest therapy in Japan has promoted the multi-functional use of forest resources and the development of tourism (80). On the one hand, this greatly reduces the pressure brought by carbon emissions to local development, and at the same time improves the completeness of the industrial chain in the circular economy model (81). Local economic development will ultimately help enterprises and residents better protect forest resources and the natural environment. The core issue for policy authorities is to constantly evaluate the effectiveness of policies to determine when appropriate policy adjustments should be made and to balance development across different regions (82). In addition, the health sector and the economic management sector have different criteria for evaluating the effectiveness of a policy. Since the objectives of promoting public health and local economic development involve cross-border collaboration between multiple government departments, continuous political and policy support from the central government level is essential.

**Figure 9:**
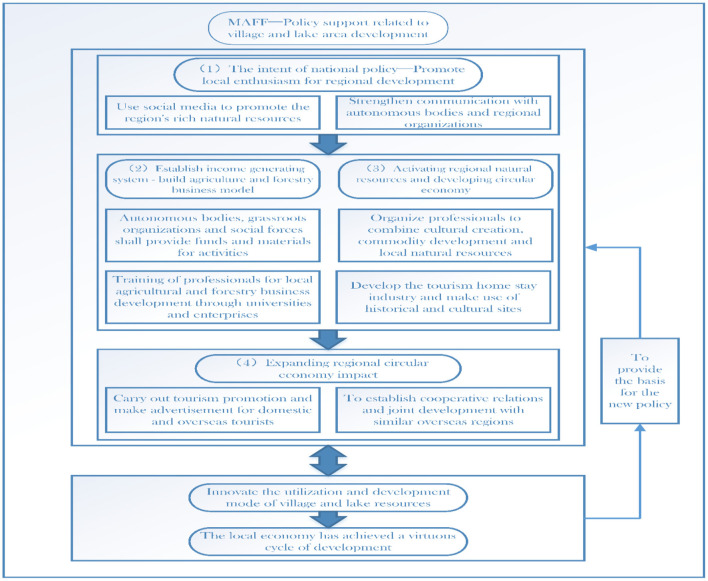
The ministry of agriculture, forestry and fisheries' leading role in the development planning of forest services.

As the most important manager of forest resources, The Ministry of Agriculture, Forestry and Fisheries plays a leading role (Figure 9) in the development planning of forest services. The policy intention of the forest service development plan led by the MAFF is to promote the enthusiasm of local governments and enterprises for the development of forest resources. The entire policy process is based on the principle of resource sharing, focusing on the use of social media to promote forest resources, and strengthening collaboration among autonomous bodies, local organizations, and enterprises. Local autonomous bodies, grassroots organizations, and social forces, including enterprises and voluntary groups, work together to provide material support for the development of forest services, cultivate relevant professionals through universities and enterprises for the development of local agriculture and forestry businesses, and form an internal circular operation model that can ensure the industry generates income.

### Government should guide, not dominate, or intervene in the market

**Figure 10:**
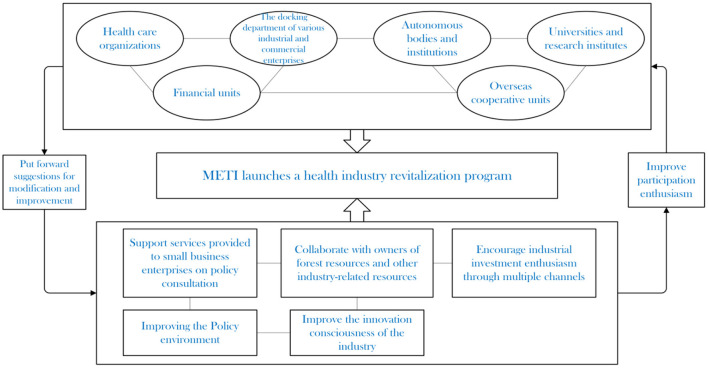
Policy support on health industry revitalization program.

The core of the forest service industry is to upgrade the traditional tourism, forestry, health care and other industries under the standard of Green, Healthy and Sustainable and develop into a comprehensive industry integrating with the forest. The Ministry of Economy, Trade and Industry is focusing on the comprehensive health industry (Figure 10) in supporting the forest service industry. METI first establishes platform for collecting opinions from various parties. As a role of supply side, it needs to improve the management system for forest service industry, optimize the investment environment and market rules, and guide industry innovation. METI provides support services to small business enterprises in business scope, policy consultation and other aspects, and cooperates with other industry-related resource owners to promote industrial investment enthusiasm through multiple channels. While introducing plans for development guidance, the leading position of enterprises in the market is respected and excessive government interference is avoided.

## Policy suggestion

### Place the forest services industry in a policy collaborative environment

**Figure 11:**
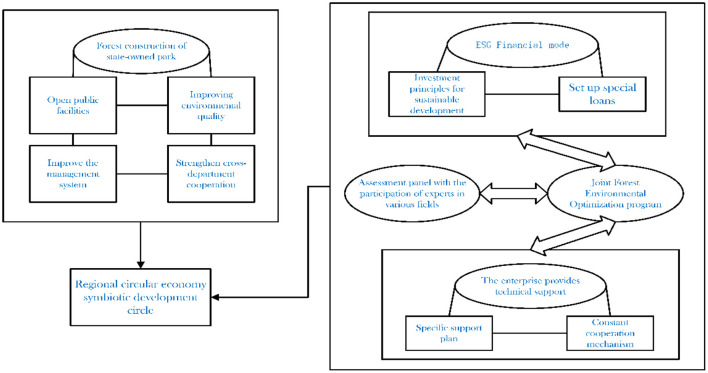
Forest services industry development in a policy collaborative environment.

In the process of developing forest resources, it is necessary to always coordinate with environmental protection and avoid the development path of first developing and then protecting (83). The government should open more public facilities (Figure 11) related to forests to create better activity spaces to attract tourists and more precisely separate tourist areas from forest reserves. In addition to promoting the integration of state-owned forest parks into the forest service industry, the government should call on experts from various fields to participate in the evaluation of the plan and guide local autonomous bodies to jointly propose the forest environment optimization plan, which is the basis of the circular economy. The new financial model ESG, which combines environmentally sustainable development with social responsibility, will provide special green loans to enterprises engaged in forest service industry, set corresponding loan issuing standards, and require enterprises to fulfill their commitments in forest environmental protection (84). The government should encourage enterprises to formulate technical plans to support forest environmental optimization, provide technical support, establish constant cooperation mechanism, promote the participation of various subjects, and improve the efficiency of forest environmental optimization.

### Forest therapy mindfulness movements go global with good policy support

Low carbon is the main path for the global response to climate. On the one hand, the movement mode of MBPs can alleviate long-term COVID-19 symptoms, and on the other hand, carbon reduction should go global to address climate change (85). Major economies should form a consensus and jointly introduce more and better policies and movement standards to encourage forest service industries worldwide, promote human health and circular economy development. In most developed and developing countries, where forest resources are plentiful, policy priorities should be focused on making forest resources better available to people's health. Scientists from different disciplines can share information, combine exercise science with energy conservation requirements, and jointly come up with more scientific standards for forest mindfulness therapy, to promote harmony between mankind and nature and help people recover more quickly from the pandemic.

## Conclusion

Under the background of over-exploitation, environmental degradation and increasing pressure on mental and physical health, how to save resources, develop economy and promote human health without over-dependence on medicines is an extremely important sustainable development policy issue. Japan has achieved great success in this area. Data from Japan suggest that forest therapy mindfulness movements are not only good for physical health but can also benefit local circular economy development when combined with a good policy environment. This should be replicated globally. The focus of public policy should be diversified and coordinated with cutting-edge ESG financial means to achieve the goal.

## Data availability statement

The original contributions presented in the study are included in the article/supplementary material, further inquiries can be directed to the corresponding author.

## Author contributions

SZ: data analysis. JT and YZ: policy analysis. HS: data collection and comparison. ZG: data ananlysis. All authors contributed to the article and approved the submitted version.

## Conflict of interest

The authors declare that the research was conducted in the absence of any commercial or financial relationships that could be construed as a potential conflict of interest.

## Publisher's note

All claims expressed in this article are solely those of the authors and do not necessarily represent those of their affiliated organizations, or those of the publisher, the editors and the reviewers. Any product that may be evaluated in this article, or claim that may be made by its manufacturer, is not guaranteed or endorsed by the publisher.
